# Construction of Conjugated Organic Polymers for Efficient Photocatalytic Hydrogen Peroxide Generation with Adequate Utilization of Water Oxidation

**DOI:** 10.3390/ma17112709

**Published:** 2024-06-03

**Authors:** Qinzhe Liu, Yuyan Huang, Yu-xin Ye

**Affiliations:** 1 Key Laboratory of Bioinorganic and Synthetic Chemistry of Ministry of Education, LIFM—Lehn Institute of Functional Materials, School of Chemistry, IGCME—Institute of Green Chemistry and Molecular Engineering, Sun Yat-sen University, Guangzhou 510275, Chinahuangyy376@mail2.sysu.edu.cn (Y.H.); 2 School of Chemical Engineering and Technology, IGCME, Sun Yat-sen University, Zhuhai 519082, China; 3 Southern Marine Science and Engineering Guangdong Laboratory (Zhuhai), Zhuhai 519082, China

**Keywords:** environmental chemistry, hydrogen peroxide, photocatalysis, water oxidation reaction

## Abstract

The visible-light-driven photocatalytic production of hydrogen peroxide (H_2_O_2_) is currently an emerging approach for transforming solar energy into chemical energy. In general, the photocatalytic process for producing H_2_O_2_ includes two pathways: the water oxidation reaction (WOR) and the oxygen reduction reaction (ORR). However, the utilization efficiency of ORR surpasses that of WOR, leading to a discrepancy with the low oxygen levels in natural water and thereby impeding their practical application. Herein, we report a novel donor–bridge–acceptor (D-B-A) organic polymer conjugated by the Sonogashira–Hagihara coupling reaction with tetraphenylethene (TPE) units as the electron donors, acetylene (A) as the connectors and pyrene (P) moieties as the electron acceptors. Notably, the resulting TPE-A-P exhibits a remarkable solar-to-chemical conversion of 1.65% and a high BET-specific surface area (1132 m^2^·g^−1^). Furthermore, even under anaerobic conditions, it demonstrates an impressive H_2_O_2_ photosynthetic efficiency of 1770 μmol g^−1^ h^−1^, exceeding the vast majority of previously reported photosynthetic systems of H_2_O_2_. The outstanding performance is attributed to the effective separation of electrons and holes, along with the presence of sufficient reaction sites facilitated by the incorporation of alkynyl electronic bridges. This protocol presents a successful method for generating H_2_O_2_ via a water oxidation reaction, signifying a significant advancement towards practical applications in the natural environment.

## 1. Introduction

With the increasing concern about environmental degradation and in situ surface water remediation, the renewable energy conversion has become a pillar supporting the advancement of sustainable development in the field of modern energy [1]. As one of the world’s 100 most used chemicals, hydrogen peroxide (H_2_O_2_) has been extensively utilized as an efficient green oxidant in chemical synthesis, wastewater treatment, paper bleaching and disinfection [2,3]. Owing to a comparable energy density to compressed hydrogen, H_2_O_2_ as a liquid fuel substitute for H_2_ is easier in storage and transportation [4]. To date, the industrial-scale production of H_2_O_2_ through the anthraquinone (AQ) oxidation process often involves drawbacks such as energy consumption and the generation of hazardous waste [5]. Therefore, it is highly desired to develop an alternative method for H_2_O_2_ manufacturing based on an efficient, affordable, green and eco-friendly process.

As a promising alternative towards sustainability, the photocatalytic production of H_2_O_2_ with remarkable efficiency and selectivity is of great interest. The ideal artificial photosynthesis of H_2_O_2_ driven by sunlight can straightforwardly exploit earth-abundant water and air as raw materials, enabling the full utilization of both the water oxidation and oxygen reduction half-reactions to activate the photocatalyst [6]. Recently, various non-metal photocatalysts, such as graphitic carbon nitride (g-C_3_N_4_) and their derivatives, resorcinol formaldehydes (RFs), as well as covalent triazine frameworks (CTFs), have been widely investigated for enhancing H_2_O_2_ photosynthesis [7,8,9]. Nonetheless, most of the reported works still encounter the following obstacles: (i) the valence band of photocatalyst cannot match the prerequisites of water oxidation reaction (WOR), resulting in only using a single mode of oxygen reduction reaction (ORR), which severely hinders the application of this technology in natural water [10,11]. (ii) Sacrificial agents such as AgNO_3_ or alcohol are employed to quench the electrons or holes, which instigates the inevitable introduction of additional substances and the generation of byproducts in the reaction system [12], and (iii) the existing photocatalysts for the synthesis of H_2_O_2_ typically have a low BET specific surface area, which obstructs the exposure of catalytic sites and is not conducive to transfer mass [13]. Moreover, preliminary investigations remain unsatisfactory due to the inadequate selectivity and unsuitable photo-redox capability to meet the stringent requirements of highly efficient full reactions for H_2_O_2_ production [14]. Accordingly, it is crucial to develop remarkable photocatalysts for overall H_2_O_2_ production via WOR and ORR dual channels with 100% atomic utilization.

Pyrene compounds are classic polycyclic aromatic hydrocarbons with a large π-conjugated plane and unique photophysical properties [15]. The easily modifiable structure of pyrene molecules and the unique planarity of the pyrene skeleton are conducive to enhancing the π-π interactions between layers, promoting the rapid separation and transfer of photo-generated charge carriers [16]. Through the donor–bridge–acceptor (D-B-A) molecular strategy containing redox moieties, the binding energy of excitons in a photocatalyst is significantly reduced, and the recombination of holes and electrons can be effectively suppressed [17].

Here, we precisely designed alternately linked electron donor–bridge–acceptor-conjugated polymeric semiconductor (termed TPE-A-P) with electron-rich tetraphenylethene moieties serving as electron donors, acetylene as the bridges and high-electron-affinity pyrene moieties acting as electron acceptors. For comparison, another photocatalyst (termed TPE-P) without alkynyl electron bridges was also successfully constructed. The activation energy of excitons in TPE-A-P photocatalyst is only 95 meV, far lower than the 141 meV for TPE-P, which was attributed to the significant role of alkynyl electron bridges. Without sacrificial agents, TPE-A-P exhibits the highest H_2_O_2_ production rate of 3028 μmol g^−1^ h^−1^ in open air, pure water, and solar light irradiation, which outperforms the majority of reported non-metallic photocatalysts. Meanwhile, the valence band potential of TPE-A-P reaches as high as 2.32 eV, giving rise to the great capacity for 2e^−^ WOR. Correspondingly, the efficiency of H_2_O_2_ production was still maintained at 1770 μmol g^−1^ h^−1^ in the absence of oxygen for TPE-A-P. Further TD-DFT calculations and in situ infrared characterization disclosed the mechanism and reactive sites of TPE-A-P and TPE-P. This study provides new insights into the molecular design of donor–bridge–acceptor photocatalysts for the full reaction of H_2_O_2_ production under anaerobic conditions.

## 2. Materials and Methods

All reagents and solvents were purchased from Sigma Aldrich or Bide pharm Co. (Shanghai, China). 

### 2.1. Synthesis of TPE-A-P

1,1,2,2-Tetrakis(4-ethynylphenyl)ethene (42.85 mg, 0.1 mmol), 1,3,6,8-tetrabromopyrene (51.78 mg, 0.1 mmol), CuI (1.9 mg, 0.1 mmol), (PPh_3_)_2_PdCl_2_ (7.02 mg, 0.1 mmol), triethylamine (10 mL) and *N*,*N*-dimethylformamide (10 mL) were added to a 100 mL reaction flask. The mixture was degassed with nitrogen for 30 min at room temperature. Then, the mixture was heated to 80 °C and refluxed for 48 h under nitrogen atmosphere. After that, the mixture was cooled to room temperature, and the precipitate was collected by filtration. The crude product was then washed with *N*,*N*-dimethylformamide, dichloromethane and ethanol. The final product was dried at 60 °C overnight to give an orange power.

### 2.2. Synthesis of TPE-P

1,1,2,2-Tetrakis(4-(4,4,5,5-tetramethyl-1,3,2-dioxaborolan-2-yl)phenyl)ethene (83.63 mg, 0.1 mmol), 1,3,6,8-tetrabromopyrene (51.78 mg, 0.1 mmol), Pd(PPh_3_)_4_ (6 mg, 0.05 mmol), *N*,*N*-dimethylformamide (10 mL) and 2 M potassium carbonate aqueous solution (1 mL) were added to a 50 mL reaction flask. The mixture was degassed with nitrogen for 30 min at room temperature. Then, the mixture was heated to 150 °C and refluxed for 48 h under nitrogen atmosphere. After that, the mixture was cooled to room temperature, and the precipitate was collected by filtration. The crude product was then washed with *N*,*N*-dimethylformamide, dichloromethane, water and ethanol. The final product was dried at 80 °C overnight to give a green power.

### 2.3. Photocatalytic Experiments

Photocatalyst (1 mg) was dispersed in 50 mL deionized water in a 100 mL beaker. After ultrasonicated for 30 min in darkness, a Xe lamp (100 mW·cm^−2^, λ > 400 nm) was used to irradiate the catalyst under magnetic stirring. At certain 30 min intervals, 1 mL of suspension was sampled and filtered with a 0.22 μm filter to remove the photocatalysts. Finally, the concentration of H_2_O_2_ was determined by using a TMB-H_2_O_2_-HRP enzymatic assay. The reaction between H_2_O_2_ and TMB was as follows:

$$H_{2}O_{2}+\mathrm{TMB}\overset{\;\mathrm{HRP}\;}{\to }H_{2}O+\mathrm{oxTMB}$$

3,3′,5,5′-Tetramethylbenzidine (TMB) solution was prepared as follows: 15 mg of TMB was dissolved in 0.3 mL of DMSO, followed by the addition of 5 mL of glycerol. Moreover, 45 mL of deionized water containing 20 mg of ethylenediaminetetraacetic acid (EDTA) and 95 mg of citric acid was added to the above solution. Finally, the solution was filled to 500 mL with deionized water. 

Horseradish peroxidase (HRP) solution was prepared as follows: 2 mg of peroxidase (from horseradish) was dissolved in 10 mL of deionized water.

Determination of the calibration curve was as follows: The standard H_2_O_2_ (0, 0.3, 0.6, 1.5, 3, 7.5 and 15 mM) solutions were prepared. Moreover, 10 μL of the above-known concentration H_2_O_2_ solution was taken, followed by the addition of 10 μL of HRP solution and 200 μL of TMB solution. After 3 min, 10 µL of concentrated hydrochloric acid was added to the above solution. The concentration of the H_2_O_2_ was calculated based on the absorbance at 450 nm by a UV–vis spectrophotometer. According to the linear relationship between signal intensity and H_2_O_2_ concentration, the H_2_O_2_ standard curve was drawn for subsequent H_2_O_2_ concentration calibration.

## 3. Results and Discussion

### 3.1. Chemical and Physical Characterization

**Figure 1:**
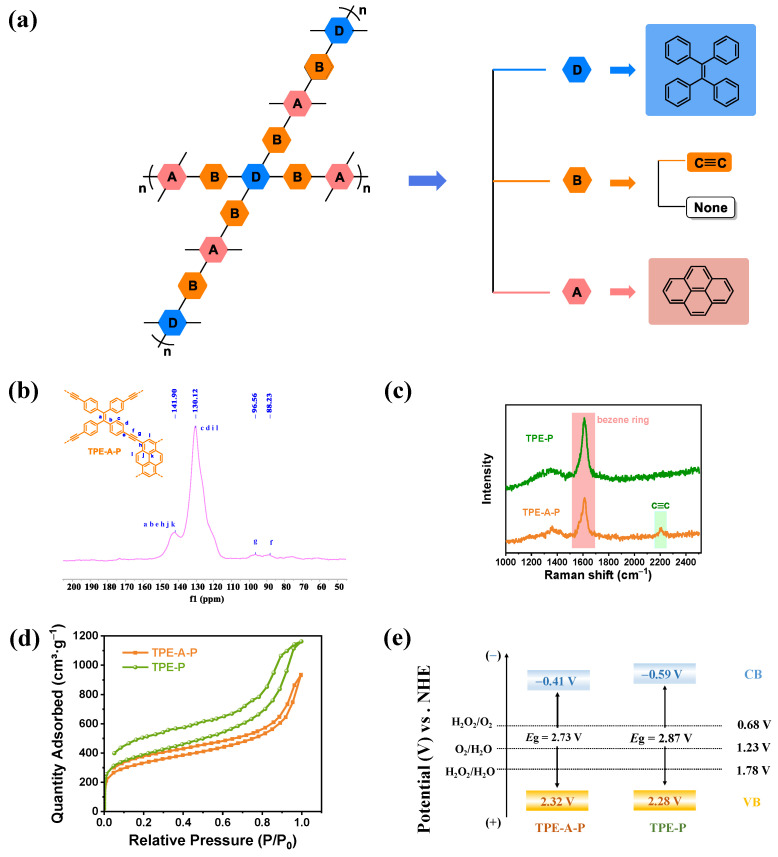
Structure and characterization of TPE-A-P and TPE-P. (**a**) Molecular structures of TPE-A-P and TPE-P. (**b**) Solid state ^13^C CP-MAS NMR spectra of TPE-A-P. (**c**) Raman spectra of TPE-A-P and TPE-P. (**d**) N_2_ adsorption–desorption isotherms of TPE-A-P and TPE-P. (**e**) Schematic illustration of the electronic structure of TPE-A-P and TPE-P.

TPE-A-P (Figure 1a) was firstly synthesized via the classic Sonogashira–Hagihara cross-coupling reaction between 1,1,2,2-tetrakis(4-ethynylphenyl)ethene (TPE) and 1,3,6,8-tetrabromopyrene (P) with acetylene as connectors, while TPE-P was prepared by the Suzuki coupling reaction between 1,3,6,8-tetrabromopyrene (P) and 1,1,2,2-tetrakis(4-(4,4,5,5-tetramethyl-1,3,2-dioxaborolan-2-yl)phenyl)ethene (TTMBPE).

The powder X-ray diffraction (XRD) patterns of target TPE-A-P and TPE-P photocatalysts displayed the features of amorphous carbon (Figure S1). The compositions and structures of both materials were further characterized by Fourier-transform infrared (FTIR) and ^13^C solid-state NMR spectroscopy (^13^C ssNMR). The peak at 3275 cm^−1^, assigned to the ≡C–H stretching vibrations from the TPE terminal alkyne, vanished in the FTIR spectra of TPE-A-P (Figure S2), indicating the successful construction of TPE-A-P. Meanwhile, the complete disappearance of the characteristic B–O peak at 1359 cm^−1^ suggested the successful preparation of TPE-P [18]. The characteristic alkynyl peak at ≈90 ppm was observed in the solid-state ^13^C NMR spectrum of TPE-A-P (Figure 1b), confirmed by the C≡C peak at 2209 cm^−1^ in the Raman spectra (Figure 1c) [19]. In contrast, no alkynyl signals were detected in those of TPE-P (Figure S3). X-ray photoelectron spectroscopic (XPS) analysis was performed to investigate the electronic structure and elemental composition of materials. As expected, Figure S4 showed that only the existence of the C element was observed in both samples. In the C 1s spectra, two peaks appearing at 283.7 and 284.6 eV can be attributed to C–C and C=C in TPE-A-P (Figure S4a), while a single peak at 284.6 eV was deconvoluted from the C 1s spectra of TPE-P (Figure S4b). Collectively, these chemical characterizations offered robust evidence supporting the successful synthesis of TPE-A-P and TPE-P [20].

Nitrogen adsorption–desorption isotherms were measured at 77 K to evaluate the porous porosities of TPE-A-P and TPE-P (Figure 1d). The Brunauer–Emmett–Teller (BET)-specific surface areas were determined to be 1132 and 1334 m^2^·g^−1^ for TPE-A-P and TPE-P, respectively. According to the IUPAC classification, both TPE-A-P and TPE-P possessed type IV adsorption–desorption isotherms, and the corresponding pore sizes of two materials illustrated in Figure S5 proved that both TPE-A-P and TPE-P possessed mesoporous properties. As shown in Figure S6, field-emission scanning electron microscope (FE-SEM) and transmission electron microscopy (TEM) revealed that two samples exhibited tightly exfoliated structures on their surfaces. Additionally, Thermogravimetric analysis (TGA) revealed that TPE-A-P possesses excellent thermal stability with a decomposed temperature above 310 °C under N_2_ atmosphere (Figure S7a).

The electronic structures of TPE-A-P and TPE-P were also of vital importance in the overall photosynthesis of H_2_O_2_. The electronic structures of TPE-A-P and TPE-P were determined by Mott–Schottky tests and valence band XPS (VB-XPS) spectra. The conduction band minima (CB_min_) of TPE-A-P and TPE-P were characterized to be −0.41 and −0.59 eV versus NHE from the Mott–Schottky tests (Figure S8). Moreover, the slopes of TPE-A-P and TPE-P were positive, indicating they were n-type semiconductors. As illustrated in Figure S9, VB-XPS spectra showed that the VB maxima (VB_max_) of TPE-A-P and TPE-P were 1.92 and 2.09 eV, respectively. The VB-XPS was used to ascertain the energy level difference from VB_max_ to Fermi level, and Kelvin probe force microscopy (KPFM) was employed to determine the energy level difference from Fermi level to vacuum level (Figure S10). Subsequently, the vacuum level to the electrode potential can be converted from the equation E_VB_ = Φ + VB_max_ − 4.44. Here, Φ is the work function of semiconductors detected by KPFM, and 4.44 is the value of the absolute potential of the standard hydrogen electrode [21]. The Φ of TPE-A-P and TPE-P were 4.84 and 4.63 eV, respectively. Consequently, the E_VB_ of TPE-A-P and TPE-P were calculated to be 2.32 and 2.28 eV. Therefore, the bandgaps of TPE-A-P and TPE-P were 2.73 and 2.87 eV. The electronic structures of TPE-A-P and TPE-P are illustrated in Figure 1e, and both photocatalysts mentioned above are thermodynamically sufficient for the photosynthesis of H_2_O_2_ through both the WOR and ORR pathways [22,23].

### 3.2. Photocatalytic Performance

**Figure 2:**
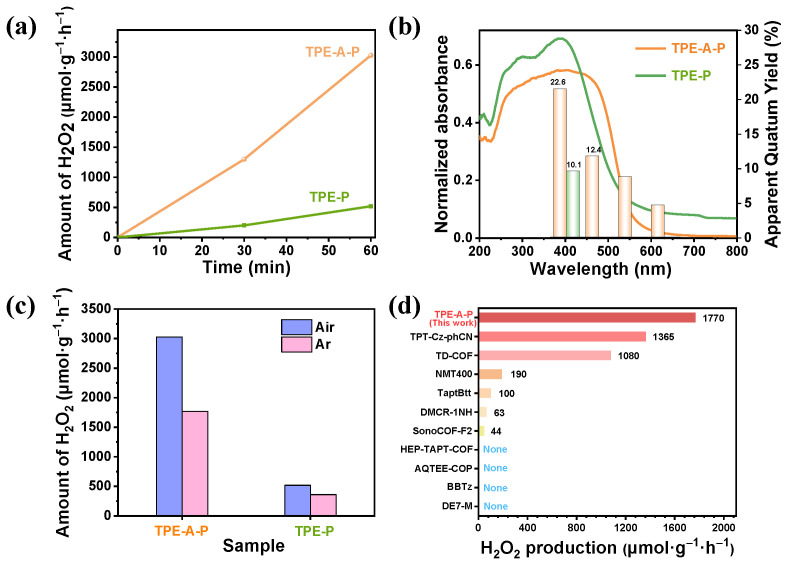
The photocatalytic performance of TPE-A-P and TPE-P for H_2_O_2_ production. (**a**) The photocatalytic H_2_O_2_ generation rate of TPE-A-P and TPE-P in open air and pure water without any additives. (**b**) UV–vis diffuse reflectance spectra of TPE-A-P and TPE-P and the AQY of TPE-A-P at 420, 470, 550 and 620 nm. (**c**) Photocatalytic production of H_2_O_2_ by TPE-A-P and TPE-P in pure water under different atmospheres. (**d**) Activity comparison between TPE-A-P and other reported photocatalysts for H_2_O_2_ production in anaerobic conditions.

The photocatalytic H_2_O_2_ production was conducted by suspending the as-synthesized TPE-A-P and TPE-P (1 mg) in pure water (50 mL) without any sacrificial agents or continuous O_2_ bubbling under Xenon-lamp light with the UV light cutoff (>400 nm, 100 mW·cm^−2^). Figure 2a showed that the photocatalytic H_2_O_2_ generation rate for TPE-A-P was 3028 μmol g^−1^ h^−1^, which was nearly six times that of TPE-P (518 μmol g^−1^ h^−1^). Remarkably, the amount of photosynthetic hydrogen peroxide remained a rapid increase within 5 h for TPE-A-P in pure water. (Figure S11). Additionally, TPE-A-P maintained its high efficiency after five consecutive cycles (Figure S12), indicating its excellent reusability and stability. By contrast, TPE-P exhibited a significant decrease in catalytic activity after four runs. The apparent quantum yield (AQY) of TPE-A-P reached 22.6%, 12.4%, 9.2% and 4.9% at 420, 470, 550 and 620 nm, respectively, compared to only 10.1% at 420 nm for TPE-P (Figure 2b). The solar–chemical-conversion (SCC) efficiency of TPE-A-P achieved 1.65%, which was about 16 times higher than the average solar-to-biomass conversion efficiency of typical plants (≈0.1%) [24,25,26,27,28]. In addition, the properties of TPE-A-P remained unchanged. The morphology and structure were maintained after 5 h irradiation (Figure S6). Notably, TPE-A-P maintained more than 50% of its activity in H_2_O_2_ generation even under anaerobic conditions (Figure 2c), which prominently surpassed photocatalysts reported in previous works listed in Figure 2d and Table S1 [29,30]. 

**Figure 3:**
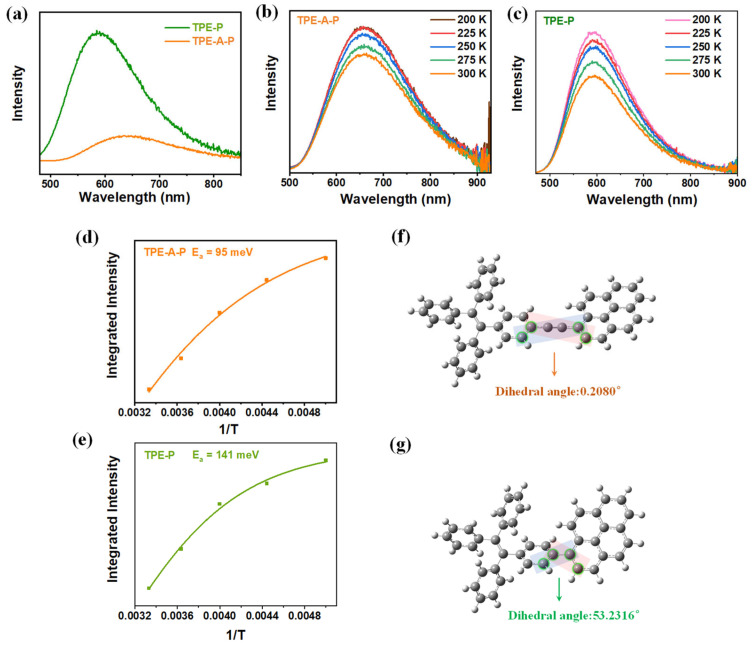
Investigation of the charge separation efficiency in two materials. (**a**) Photoluminescence spectra of TPE-A-P and TPE-P. Temperature-dependent PL spectra for (**b**) TPE-A-P and (**c**) TPE-P. The exciton activation energy of (**d**) TPE-A-P and (**e**) TPE-P. The dihedral angles of (**f**) TPE-A-P and (**g**) TPE-P.

Our investigation proceeded to explore the reason behind the remarkable photosynthetic efficiency. Figure S13 showed that the photocurrent density of TPE-A-P was significantly higher than that of TPE-P, which suggested the availability of more light-induced carriers in the photocatalytic reaction. Electrochemical impedance spectroscopy (EIS) measurement was also conducted to explore the interfacial charge transfer resistance of TPE-A-P and TPE-P (Figure S14). According to the Nyquist plots, the smaller semicircle diameter of TPE-A-P indicated the excellent separation of photogenerated electrons and holes, as well as a faster interfacial charge transfer in TPE-A-P. From the steady-state photoluminescence (PL) emission spectra (Figure 3a), the overall recombination efficiency of excitons was relatively low in TPE-A-P, whereas they were still significant in TPE-P, suggesting that the exciton radiative recombination was greatly suppressed in TPE-A-P. Temperature-dependent PL spectroscopy was also conducted to investigate the charge migration dynamics (Figure 3b,c). The exciton activation energy (E_a_) of the photoluminescence quenching process was estimated by the Arrhenius equation (Figure 3d,e) [31]. The E_a_ derived were 95 meV for TPE-A-P and 141 meV for TPE-P. We believed that the lower E_a_ of TPE-A-P indicated much easier separation of excitons than TPE-P. In addition, the dihedral angles of TPE-A-P and TPE-P were 0.218° and 53.23°, respectively (Figure 3f,g) [32]. The introduction of acetylene as the bridge greatly facilitated the coplanarity of the electron acceptors and electron donors, which was conducive to the charge transfer and exciton dissociation process in the D-B-A structure of TPE-A-P.

### 3.3. Pathways for Photosynthesis of H_2_O_2_

**Figure 4:**
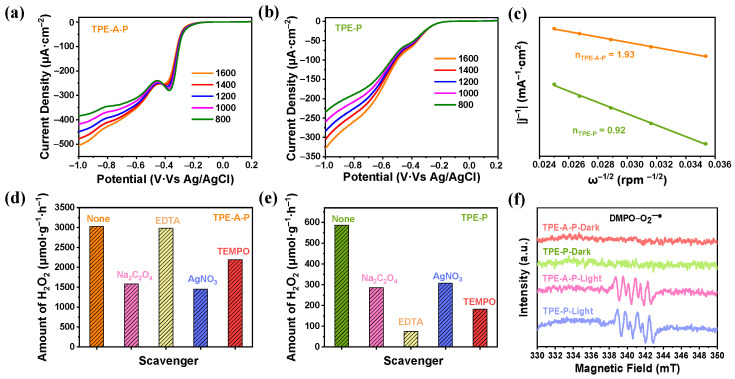
Investigation of pathways for ORR process. Linear-sweep RDE voltammograms of (**a**) TPE-A-P and (**b**) TPE-P. (**c**) Koutecky–Levich plots obtained from RDE measurements. Photosynthesis of H_2_O_2_ with different scavengers of (**d**) TPE-A-P, (**e**) TPE-P. (**f**) EPR spectra of DMPO-O_2_^−•^ for TPE-A-P and TPE-P.

Control experiments and electrochemical measurements were conducted to investigate the pathways for generating H_2_O_2_ in photocatalysis. Rotating disk electrode (RDE) voltammetry was executed to explore the average electron transfer number in the ORR process for two materials. The linear sweep voltammetry curves were acquired with different rotating speeds (Figure 4a,b). The Koutecky–Levich (K–L) plots in Figure 4c revealed that the average electron transfer number (n) of TPE-A-P and TPE-P were 1.93 and 0.92, respectively, suggesting varying degrees of selectivity of ORR process when TPE-A-P and TPE-P were utilized [33]. The number of 1.93 was close to the theoretical value for directly and selectively reducing O_2_ into H_2_O_2_ for TPE-A-P, while 0.92 indicated single electron transfer occurred in the presence of TPE-P. In addition, a ring-disk electrode (RRDE) was also conducted to investigate the selective electron transfer during ORR. As shown in Figure S15a, the ring current of TPE-A-P exhibited a rapid enhancement along with the decrease in potential, implying a greater generation of H_2_O_2_ from the disk part. The n values of TPE-A-P and TPE-P were then calculated to be 2.1 and 1.1, respectively (Figure S15b). In order to further probe the differences in the ORR process, we conducted a series of sacrificial agent experiments (Figure 4d,e). With the existence of 2,2,6,6-tetramethyl-1-piperidinyl oxy (TEMPO) as O_2_^−•^ sacrificial agents, the yield of photosynthetic H_2_O_2_ for TPE-A-P decreased slightly, while a noticeable reduction for TPE-P was observed. This result revealed a different majority of intermediates within the ORR process between the two materials. Combined with the RDE test results, TPE-A-P was more inclined to undergo a direct 2e^−^ one-step oxygen reduction (O_2_ → H_2_O_2_) to produce H_2_O_2_. Conversely, TPE-P tended to undergo an indirect sequential 1e^−^ two-step oxygen reduction (O_2_ → O_2_^−•^ → H_2_O_2_) [34], where O_2_ is first singly reduced to form O_2_^−•^, and then the O_2_^−•^ acquires another electron to generate H_2_O_2_. Unfavorably, O_2_^−•^ is easily quenched and can attack the generated H_2_O_2_, causing the decomposition of H_2_O_2_ and thus resulting in a decrease in yield [35,36]. Therefore, the 2e^−^ one-step oxygen reduction is more advantageous to the efficiency of the ORR process in photocatalytic H_2_O_2_ production. The 2e^−^ one-step ORR selectivity of TPE-A-P guaranteed an exceeding performance. When using EDTA-2Na as a hole scavenger, TPE-A-P exhibited a tiny decrease in the photocatalytic yields of H_2_O_2_ and in sharp contrast to TPE-P, which further confirmed a better ORR performance for TPE-A-P. Electron paramagnetic resonance (EPR) experiments were performed to detect reactive oxygen species (ROS). Figure 4f demonstrated that the presence of O_2_^−•^ was detected in both TPE-A-P and TPE-P systems with 5,5-dimethyl-1-pyrroline *N*-oxide (DMPO) as the spin-trap agent. These results collectively indicated that TPE-A-P followed both a 1e^−^ two-step ORR and 2e^−^ one-step ORR pathways for H_2_O_2_ production, while TPE-P mainly followed a 1e^−^ two-step ORR pathway. 

**Figure 5:**
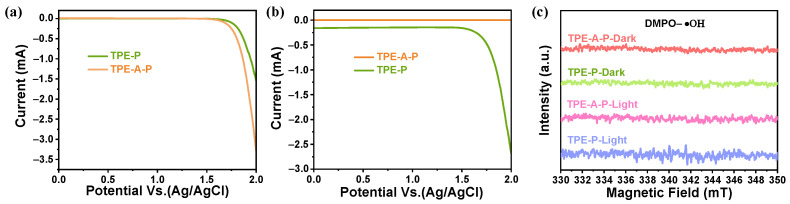
Studies of pathways for WOR process. Rotating ring-disk electrode voltammograms obtained in 0.1 M phosphate buffer solution with a rotation rate of 1600 rpm. (**a**) The Pt ring was set at 0.6 V to detect H_2_O_2_. (**b**) The Pt ring was set at −0.23 V to detect O_2_. (**c**) EPR spectra of DMPO-·OH for TPE-A-P and TPE-P.

On the other hand, the WOR process was also vital for the photosynthesis of H_2_O_2_ since both TPE-A-P and TPE-P maintained more than one-half of the activities in an Ar atmosphere. Correspondingly, both materials were capable of generating H_2_O_2_ with almost half of the efficiency when silver nitrate (AgNO_3_) served as an electron sacrificial agent, confirming an appreciable capacity of WOR (Figure 4d,e). The RRDE tests under nitrogen atmosphere were utilized to determine the products of WOR. TPE-A-P and TPE-P displayed substantial reduction currents at the Pt ring electrode when the applied potentials were set at 0.6 V (Figure 5a), which were identified as the reduction of H_2_O_2_ [37]. Noticeably, TPE-A-P exhibited better 2e^−^ WOR performance than TPE-P. Only TPE-P showed distinct reduction currents when the applied potentials were set at −0.23 V (Figure 5b), which was attributed to the reduction of O_2_. This revealed that TPE-P followed both 2e^−^ and 4e^−^ WOR pathways, while TPE-A-P mainly followed a 2e^−^ WOR pathway, indicating an excellent selectivity favoring the photosynthesis for H_2_O_2_. In addition, H_2_O_2_ could also be photosynthesized through indirect 2e^−^ water oxidation, which involved hydroxyl radicals (·OH) as the intermediate. EPR experiments revealed no detectable ·OH signals by using DMPO as a trapping agent under both light and dark conditions (Figure 5c), indicating no indirect 2e^−^ pathway of water oxidation for both photocatalysts [38]. Consequently, TPE-A-P and TPE-P facilitated the formation of H_2_O_2_ via the direct 2e^−^ pathway of WOR.

### 3.4. Active Sites for Both ORR and WOR

**Figure 6:**
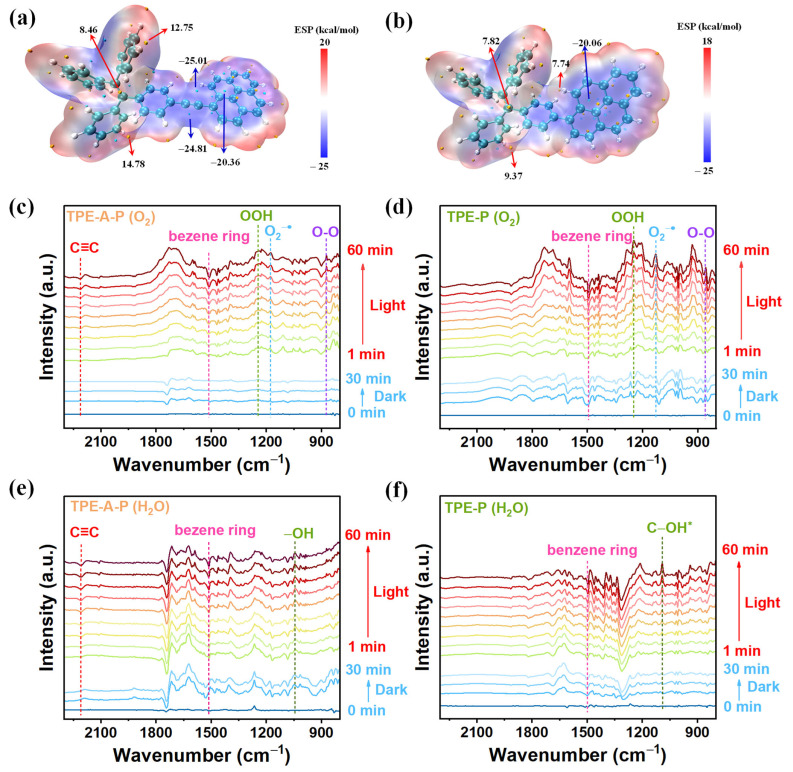
Active sites of TPE-A-P and TPE-P for H_2_O_2_ production. Electrostatic potential maps for (**a**) TPE-A-P and (**b**) TPE-P. The yellow dots represent the points of maximum surface potential, and the green dots represent the points of minimum surface potential. In situ DRIFTS spectra of (**c**) TPE-A-P under O_2_, (**d**) TPE-P under O_2_, (**e**) TPE-A-P under H_2_O, (**f**) TPE-P under H_2_O.

To have a clear insight into the mechanism of photocatalytic H_2_O_2_ generation, we conducted theoretical studies based on the model compounds of TPE-A-P and TPE-P. As shown in Table S2, the dominant transition of TPE-A-P and TPE-P was both *S*_0_ → *S*_1_ with the largest values of oscillator strengths [39]. Therefore, time-dependent density functional theory (TD-DFT) in terms of the transition of *S*_0_ → *S*_1_ was exploited to investigate the active sites responsible for the photosynthesis of H_2_O_2_ (Figure S16) [40]. Accordingly, the photoinduced holes of excited TPE-A-P and TPE-P were primarily located at the TPE sites, while the photoinduced electrons were situated at the pyrene sites. Moreover, the electrostatic potential maps in Figure 6a,b depicted the electrical distribution of TPE-A-P and TPE-P, indicating a prominent negative electron charge surface with blue color on pyrene moieties as well as a significant positive charge surface with red color on the TPE sites for both materials. The distribution of the negative and positive charges was compatible with the locations of photoinduced holes and electrons. TPE-A-P possessed unique electron-rich alkynyl electron bridges, which were capable of transferring electrons to oxygen, implying acetylene moieties as the active sites for ORR [41]. Additionally, TPE-A-P exhibited a higher density of positive charges on the TPE sites in comparison to TPE-P. This characteristic facilitated the separation of charges, enabling TPE-A-P to efficiently catalyze the synthesis of H_2_O_2_ from water oxidation.

In situ diffuse reflectance infrared Fourier transform spectroscopy (DRIFTS) measurements of TPE-A-P and TPE-P were tested to demonstrate the active sites for ORR and WOR. As exhibited in Figure 6c,d, under a continuous steam-saturated O_2_ flow and when the system was irradiated for 60 min, new vibrations corresponding to O–O (855 cm^−1^), O_2_^−•^ (1129 cm^−1^), OOH (1246 cm^−1^) for TPE-P emerged owing to the formation of intermediates from the 1e^−^ two-step ORR process [42]. For TPE-A-P, vibrations corresponding to O–O (874 cm^−1^), O_2_^−•^ (1179 cm^−1^), OOH (1245 cm^−1^) also appeared. However, the variation in the intensity of these signals for TPE-A-P was relatively low, implying a different selectivity for ORR pathways. It is noteworthy that the peak intensity corresponding to C≡C (2209 cm^−1^) showed a gradual enhancement after illumination, which elaborated alkynyl electron bridges served as active sites in the ORR reaction. Furthermore, the characteristic signals attributed to the benzene rings (1516 cm^−1^ for TPE-A-P and 1497 cm^−1^ for TPE-P) gradually intensified under irradiation, which further confirmed the benzene rings as active sites in the ORR process [43]. In combination with the calculation results of hole and electron contribution rates in TD-DFT, the benzene rings located at pyrene moieties were involved in H_2_O_2_ production via ORR reaction.

The active sites for WOR were further demonstrated by in situ IR spectrometry measurements after a continuous steam-saturated H_2_O (Figure 6e,f). Under light irradiation, the formation of a new infrared signal corresponding to –OH (1045 cm^−1^ for TPE-A-P) and C–OH^*^ (1092 cm^−1^ for TPE-P) evidenced the adsorption of H_2_O and subsequent oxidation reactions [44]. Additionally, the enhancement of the characteristic peak of C≡C (2209 cm^−1^ for TPE-A-P) during the illumination process indicated the involvement of acetylene sites to produce H_2_O_2_ via WOR reaction. Moreover, the peak at 1513 cm^−1^ for TPE-A-P and 1483 cm^−1^ for TPE-P, assigned to the vibration of benzene rings, showed a rising signal intensity [45]. Binding with the analysis of the distribution of the photoinduced holes in TD-DFT, the benzene rings situated at TPE moieties served as active sites for the WOR reaction.

These results emphasized variation in active sites for WOR and ORR between TPE-A-P and TPE-P. Based on the experimental and computational results, the primary mechanism of TPE-A-P photocatalytic H_2_O_2_ production was proposed as follows: under visible-light illumination, the photoinduced electrons were primarily trapped at the pyrene sites, whereas the holes predominantly collected on the TPE units. Particularly due to the capacity of adsorbing both water and oxygen, alkynyl electron bridges served as active sites for both WOR and ORR. These led to the production of H_2_O_2_ through a direct 2e^−^ one-step ORR on the pyrene and acetylene moieties as well as a direct 2e^−^ WOR pathway at TPE and acetylene sites. 

## 4. Conclusions

In summary, we synthesized an effective D-B-A conjugated organic polymer for photosynthetic H_2_O_2_. The photosynthetic rate of H_2_O_2_ reaches 3028 μmol g^−1^ h^−1^ under ambient conditions. More importantly, the activity was maintained for more than one-half, even under argon conditions. Our investigations show that the incorporation of alkynyl electron bridges can effectively diminish exciton excitations, thereby suppressing the radiative recombination of excitons. Meanwhile, the differences in the ORR pathways between TPE-A-P and TPE-P lead to a gap of performance in photocatalytic H_2_O_2_ production, where TPE-A-P mainly exhibited a 2e^−^ one-step ORR pathway and TPE-P followed a 1e^−^ two-step pathway. Additionally, TPE-A-P exhibited direct 2e^−^ water oxidation selectivity and better performance in WOR than TPE-P. In addition to possessing TPE as the active sites for water oxidation reaction and pyrene units as the sites for oxygen reduction reaction in both materials, the acetylene groups in TPE-A-P also participate in both WOR and ORR process, leading to an overall increase in H_2_O_2_ production performance. This work introduces a novel D-B-A protocol for the photocatalytic production of H_2_O_2_ in oxygen-limited conditions and holds an important implication for natural water remediation. 

## Data Availability

The raw data supporting the conclusions of this article will be made available by the authors on request.

## Supplementary Materials

The following supporting information can be downloaded at: https://www.mdpi.com/article/10.3390/ma17112709/s1, Figure S1: PXRD patterns of TPE-A-P and TPE-P; Figure S2: FTIR of TPE-A-P, TPE-P, TPE and TTMBPE; Figure S3: Solid state ^13^C CP-MAS NMR spectra of TPE-P; Figure S4: C(1s) spectra of (a) TPE-A-P, (b) TPE-P; Figure S5: The pore size distributions of (a) TPE-A-P, (b) TPE-P; Figure S6: SEM images of (a) TPE-A-P, (b) TPE-A-P after 5-h irradiation, (c) TPE-P, (d) TPE-P after 5-h irradiation. TEM images of (e) TPE-A-P, (f) TPE-A-P after 5-h irradiation, (g) TPE-P, (h) TPE-P after 5-h irradiation; Figure S7: Thermogravimetric analysis (TGA) profiles of (a) TPE-A-P and (b) TPE-P ranging from room temperature to 800 °C at 10 °C·min^−1^; Figure S8: Mott Schottky plots of (a) TPE-A-P, (b) TPE-P; Figure S9: VB-XPS spectra of (a) TPE-A-P, (b) TPE-P; Figure S10: KPFM images of (a) HOPG, (b) TPE-A-P, (c) TPE-P; Figure S11: Photosynthetic amount of H_2_O_2_ generation from TPE-A-P and TPE-P in pure water under 5-h irradiation; Figure S12: Cycle experiments of TPE-A-P and TPE-P in deionized water; Figure S13: Current-Time (I-t) curves under visible light illumination of (a) TPE-A-P and TPE-P in Ar, (b) TPE-A-P and TPE-P in O_2_; Figure S14: Nyquist plot of TPE-A-P and TPE-P; Figure S15: (a) ORR polarization curves of photocatalysts on RRDE at 1600 rpm in O_2_-saturated phosphate buffer solution. (b) The corresponding number (n) of electron transfer as a function of the applied potential; Figure S16: The distribution of electrons and holes in excited state of (a) TPE-A-P and (b) TPE-P; Table S1: The efficiency comparison between TPE-A-P and previous reported photocatalysts for H_2_O_2_ production in anaerobic conditions; Table S2: Oscillator strengths of excited states of monomers from TPE-A-P and TPE-P. References [6,30,42,46,47,48,49,50,51,52,53,54,55,56,57,58,59,60,61,62,63,64,65,66,67] are cited in the Supplementary Materials.
