# Supplementary material for: Construction of Conjugated Organic Polymers for Efficient Photocatalytic Hydrogen Peroxide Generation with Adequate Utilization of Water Oxidation
Source: Materials (Basel). 2024 Jun 3;17(11):2709. doi: 10.3390/ma17112709 (PMC11173575; doi:10.3390/ma17112709)
Supplement: Supplementary file 1 [file materials-17-02709-s001.zip › materials-2999813-supplementary.pdf]

Supplementary Material

Construction of conjugated organic polymers for efficient photocatalytic hydrogen peroxide generation with adequate utilization of water oxidation

Qinzhe Liu,[1,†] Yuyan Huang,[1,†] Yu-Xin Ye,[2,3,*]

[1] Key Laboratory of Bioinorganic and Synthetic Chemistry of Ministry of Education, LIFM, School of Chemistry, IGCME, Sun Yat-sen University, Guangzhou 510275, China

[2] School of Chemical Engineering and Technology, IGCME, Sun Yat-sen University, Zhuhai 519082, China

[3] Southern Marine Science and Engineering Guangdong Laboratory (Zhuhai), Zhuhai, Guangdong 519082, China

[†] These authors contributed equally to this work.

*Correspondence:

yeyuxin5@sysu.edu.cn

1. Characterization and Methods

Powder X-ray diffraction (XRD) patterns were recorded on a powder X-ray diffraction instrument (D-MAX 2200 VPC) at 40 kV and 26 mA. Fourier transform infrared spectra (FTIR) were recorded on a PerkinElmer Frontier spectrometer. X-ray photoelectron spectroscopy (XPS) measurements were recorded using an ESCALab 250 spectrometer. UV-visible spectroscopy (UV-Vis) were obtained by a Shimadzu UV-3600 spectrometer. Solid state ^{13}C NMR spectra were recorded on CyroProbe TCT 600 MHZ. Raman spectra were conducted by using a Laser Microscopic Confocal Raman Spectrometer (Renishaw in Via) at 325 nm. Photoluminescence (PL) spectra were obtained using a FLS 1000 spectrometer. SEM images were conducted on a SU8010 emission scanning electron microscope operated at 10 kV. TEM images were studied by using JEM-ARM200P at 120 kV. N_2 isotherms and surface area measurements were determined on a JW -BK200C instrument.

1.1 SCC Efficiency Measurements

An AM 1.5 G solar simulator was employed as the light source (100 mW·cm^{-2}) to determine the SCC efficiency by photocatalytic experiments. The reaction was conducted in 100 mL deionized water with 100 mg photocatalyst in a glass bath. The SCC efficiency (η) was calculated by following equation:

$$\eta(\%) = \frac{\Delta G H_2O_2 \times nH_2O_2}{t_{ir} \times S_{ir} \times I_{AM}} \times 100\%$$

where ΔGH_2O_2 is the free energy for H_2O_2 generation (117 kJ·mol^{-1}), nH_2O_2 is the amount of H_2O_2 generated, and t_{ir} is the irradiation time (3600 s). The overall irradiation intensity (I_{AM}) of the AM 1.5 global spectrum (300 nm to 2500 nm) is 100 mW·cm^{-2}, and the irradiation area (S_{ir}) is 3.14×10^{-4} m^2.

1.2 Apparent Quantum Efficiency Analysis

The photocatalytic reaction was measured in pure deionized water (50 mL) with photocatalyst (5 mg) in a quartz tube. The tube was irradiated by a Xe lamp for 1 hour under magnetic stirring. The number of incident photons (M) was calculated by the following equation:

$$M = \frac{E\lambda}{hc}$$

In the equation, E, λ, h, and c are the average intensity of irradiation, the wavelength of the irradiation, the Planck constant, and the speed of light, respectively. The quantum efficiency was calculated by the following equation:

$$AQY = \frac{2 \times \ number\ of\ evolved\ H_2O_2\ molecules}{M} \times 100\%$$

1.3 Determination of H_2O_2 Concentration

The concentration of H_2O_2 was determined by a TMB-H_2O_2-HRP enzymatic assay. The reaction between H_2O_2 and TMB was as follow:

$$H_2O_2 \quad + \quad TMB \xrightarrow{HRP} H_2O \quad + \quad oxTMB$$

3,3′,5,5′-tetramethylbenzidine (TMB) solution: 15 mg TMB was dissolved in 0.3 mL DMSO, followed by adding 5 mL glycerol and 45 mL deionized water containing 20 mg

ethylenediaminetetraacetic acid (EDTA) and 95 mg citric acid. Then the solution was filled to 500 mL with deionized water.

Preparation of HRP solution was as follows: 2 mg of peroxidase (from horseradish) was dissolved in 10 mL of deionized water.

Determination of the calibration curve was as follows: TMB and HRP were added into the H_2O_2 solution with a known concentration. After 3 min, 10 μL concentrated hydrochloric acid was added into the solution and the measured by UV-visible spectroscopy at 450 nm. According to the liner relationship between signal intensity and H_2O_2 concentration, the H_2O_2 concentration of the samples could be determined.

1.4 Photoelectrochemical and electrochemical measurements

All measurements were conducted on electrochemical workstation (CHI 760E Instruments or CHI 760E Instruments) in a three-electrode cell system. Standard three-electrode setup consisted of Pt-wire and Ag/AgCl as counter and reference electrodes. A 300 W Xe lamp was used as irradiation source.

The samples were prepared by adding 5 mg catalyst into the solution of 180 μL ethanol and 20 μL 5 % nafion. The mixture was then dispersed by ultrasonication for 30 min.

1.5 Photocurrents measurements

The FTO glasses deposited with photocatalysts were used as working electrode. After the systems were purged with O_2 or Ar for 15 min, the measurements were performed on 0.1 M Na_2SO_4 solution.

1.6 Rotating disk electrode (RDE) measurements

A glassy carbon rotating disk electrode was employed as working electrode The electron transfer number for oxygen reduction reaction (ORR) was carried in an O_2-saturated 0.1 M phosphate buffer solution and different rotating speeds after O_2 bubbling for 30 min. The average of electron number was estimated by using Koutecky-Levich equation:

$$\frac{1}{J} = \frac{1}{J_L} + \frac{1}{J_K} = \frac{1}{B\omega^{\frac{1}{2}}} + \frac{1}{J_K}$$

$$B = 0.2nFV^{-\frac{1}{6}}CD^{\frac{2}{3}}$$

Where J is the current intensity, J_L and J_k are the kinetic and diffusion-limiting current densities, ω is the angular velocity, n is transferred electron number, F is Faraday constant (96485 $C \cdot mol^{-1}$), ν is the kinetic viscosity of water (0.01 $cm^2 \cdot s^{-1}$), C is the bulk concentration of O_2 in water (1.26×10^{-3} $mol \cdot cm^{-3}$), and D is the diffusion coefficient of O_2 (2.7×10^{-5} $cm^2 \cdot s^{-1}$).

1.7 Rotating ring-disk electrode (RRDE) measurements

1.7.1 Selective electron transfer during ORR

A ring-disk electrode was used as working electrode in a 0.1 M phosphate buffer solution (pH=7). The number of the transferred electrons was calculated following:

$$n = \frac{4I_d}{I_d + \frac{I_r}{N}}$$

where I_d is the disc current, I_r is the ring current, and N is the collection efficiency. The disk potential E_{disk} (E_{RHE}) is scanned from 1 V to -1 V at a scan rate of 10 $mV \cdot s^{-1}$, the ring potential E_{ring} is fixed to 1.2 V.

1.7.2 Products of WOR

A ring-disk electrode was used as working electrode in a 0.1 M phosphate buffer solution (pH=7) under Ar atmosphere at room temperature. After the phosphate buffer solution was purged with Ar for 30 min, the measurements were conducted with the potential of ring electrode was set to −0.23 and 0.6 V (vs. Ag/AgCl) to detect O_2 and H_2O_2, respectively.

1.8 Electron paramagnetic resonance (EPR) measurements

5,5-dimethyl-1-pyrroline *N*-oxide (DMPO) was used as a spin-trapping reagents to detect ·OH and $O_2^{-•}$. The measurements were conducted as follows: For $O_2^{-•}$ test, 1 mg catalyst was dispersed in methanol (200 μL) containing DMPO (10 μL) and O_2 was bubbled into the solution for 3 minutes. For ·OH test, catalyst (1 mg) was dispersed in deionized water (200 μL) containing DMPO (10 μL).

1.9 In situ DRIFTS measurements

A Nicolet 5700 FT-IR spectrometer was used for the in situ diffuse reflectance infrared Fourier transform spectroscopy. The cell was equipped with a KBr window. The samples were degassed at 100 °C for 3 h. After that, the samples were exposed to O_2 or H_2O vapor for 30 min in darkness to reach adsorption-desorption equilibrium. Then, the in situ DRIFTS spectra were obtained under irradiation for 60 min.

1.10 Computational methods

The Density functional theory (DFT) calculation was conducted as implemented in the Gaussian 09 D.01 program package [1] and Grimme-D3 dispersion correction was used [2]. GaussView6 was employed for visualization [3]. The geometry optimization and frequency analysis were performed at the B3LYP/6-31G(g,d) level of theory [4]. The optimized structures were utilized to calculate single-point energy with PBE0/ma-TZVP [4–6]. Time-dependent density functional theory (TD-DFT) was carried out at the PBE0/6-31G(d,p) level of theory and applied to the investigation of the transfer direction of electrons [7–9]. Analysis and visualization of hole and electron distribution were performed by Multiwfn [10,11]. Exciton binding energy was calculated by the def2-SVP basis set [12]. The electrostatic potential and Mulliken electronegativity involved in the analysis were evaluated by Multiwfn based on an efficient algorithm and filled with colors using VMD1.9.3 [10,13–15].

2. Supplementary Figures and Tables

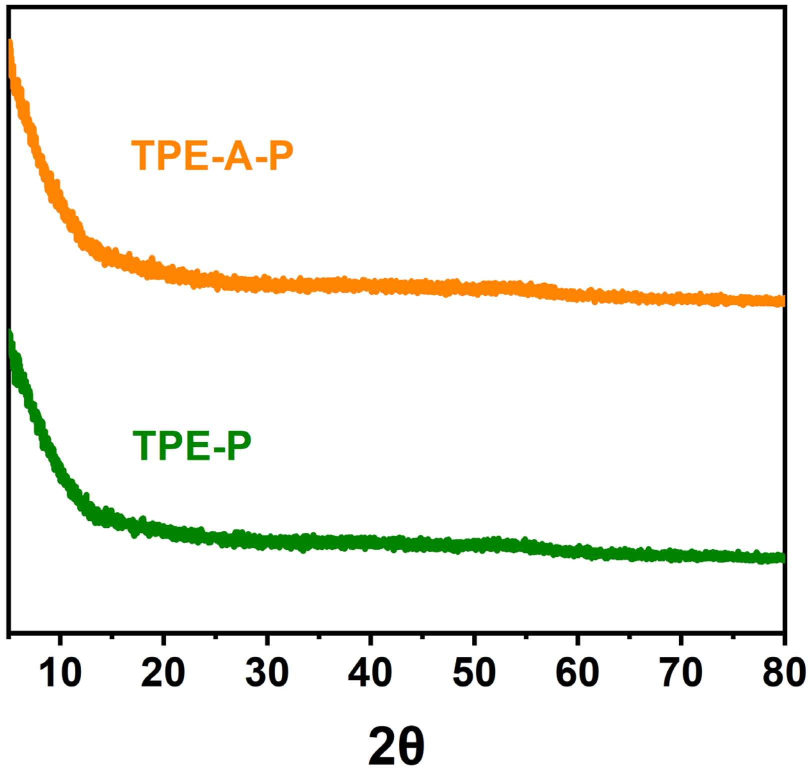

Figure S1. PXRD patterns of TPE-A-P and TPE-P.

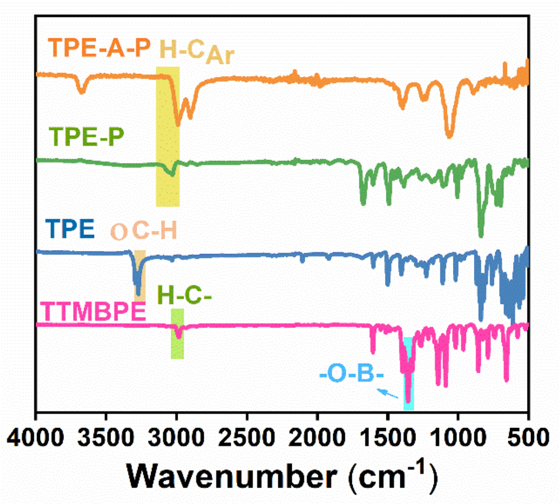

Figure S2. FTIR of TPE-A-P, TPE-P, TPE and TTMBPE.

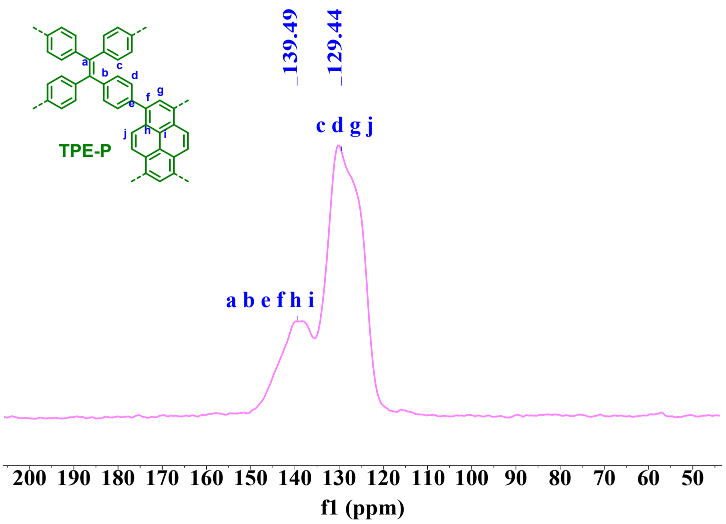

Figure S3. Solid state ^{13}C CP-MAS NMR spectra of TPE-P.

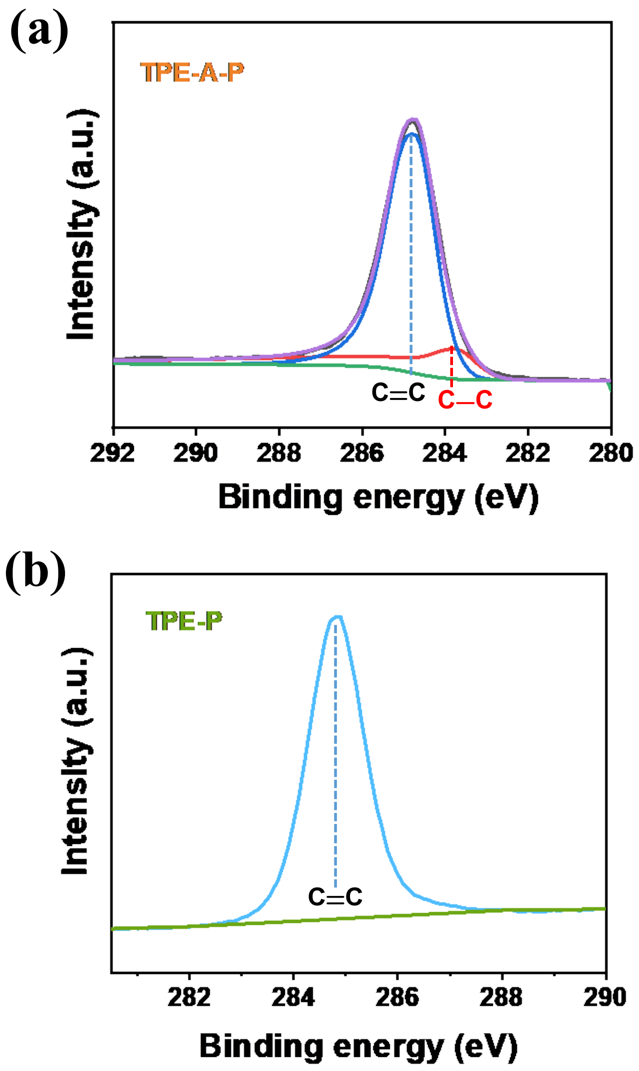

Figure S4. C(1s) spectra of (a) TPE-A-P, (b) TPE-P.

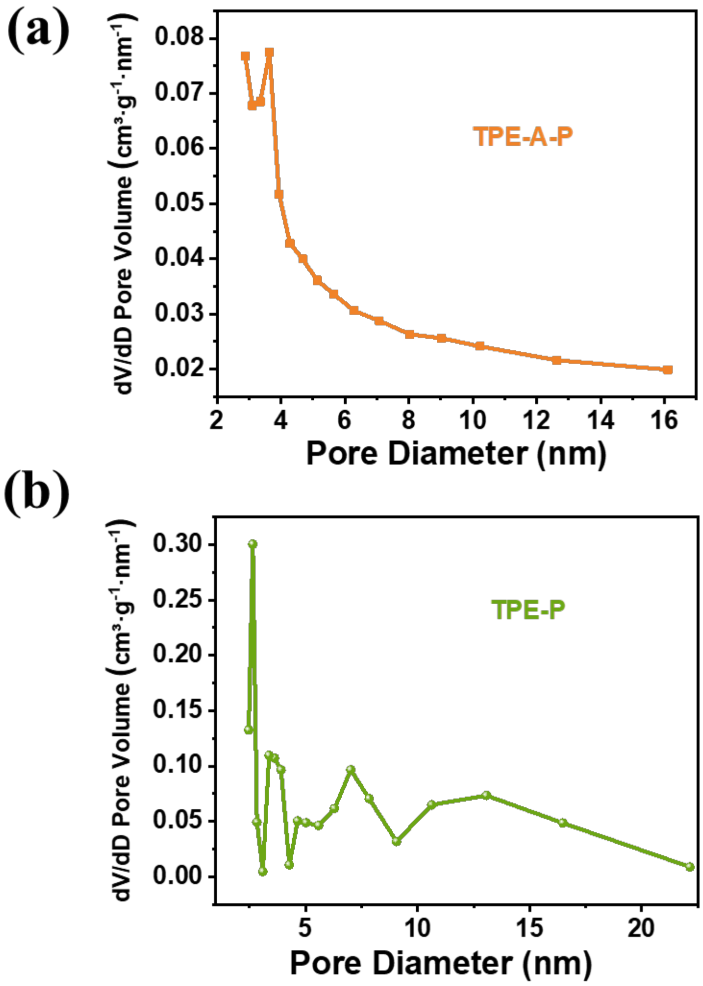

Figure S5. The pore size distributions of (a) TPE-A-P, (b) TPE-P.

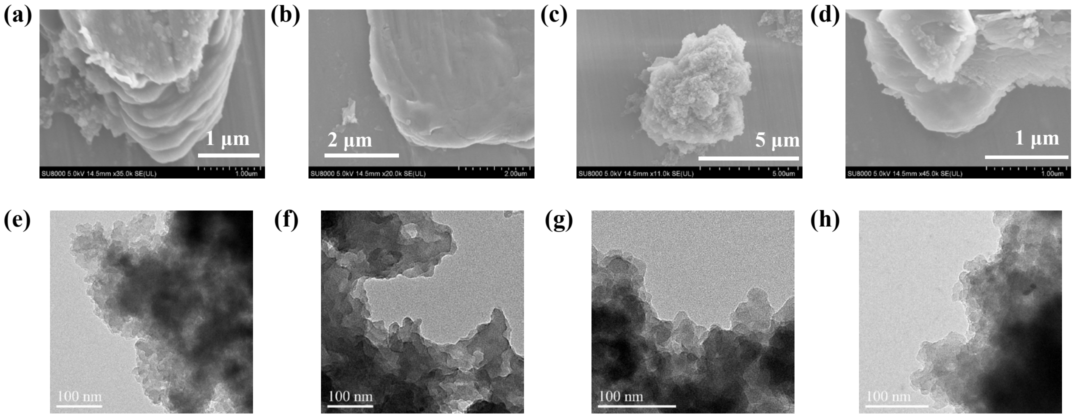

Figure S6. SEM images of (a) TPE-A-P, (b) TPE-A-P after 5-hour irradiation, (c) TPE-P, (d) TPE-P after 5-hour irradiation. TEM images of (e) TPE-A-P, (f) TPE-A-P after 5-hour irradiation, (g) TPE-P, (h) TPE-P after 5-hour irradiation.

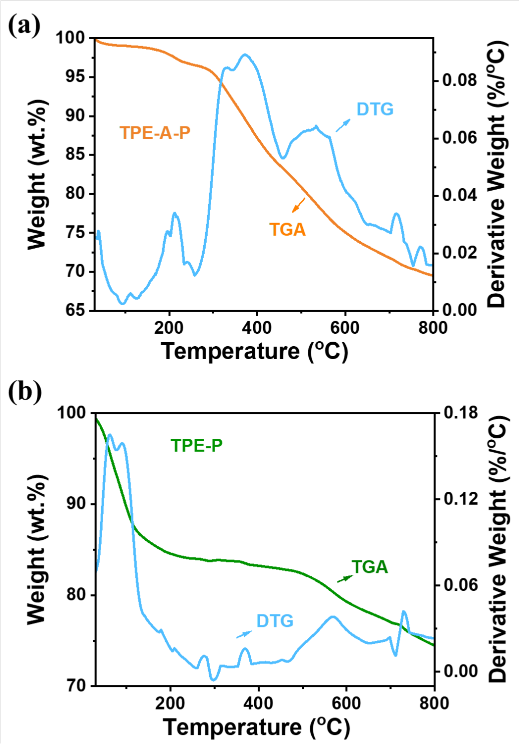

Figure S7. Thermogravimetric analysis (TGA) profiles of (a) TPE-A-P and (b) TPE-P ranging from room temperature to 800 °C at 10 °C·min^{-1}.

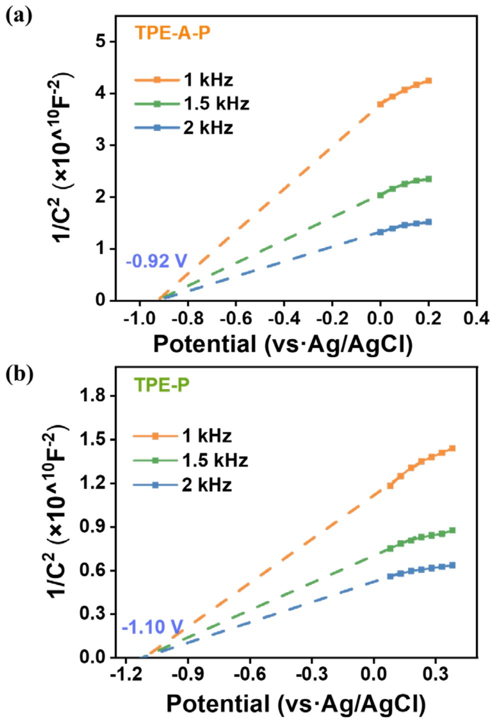

Figure S8. Mott Schottky plots of (a) TPE-A-P, (b) TPE-P.

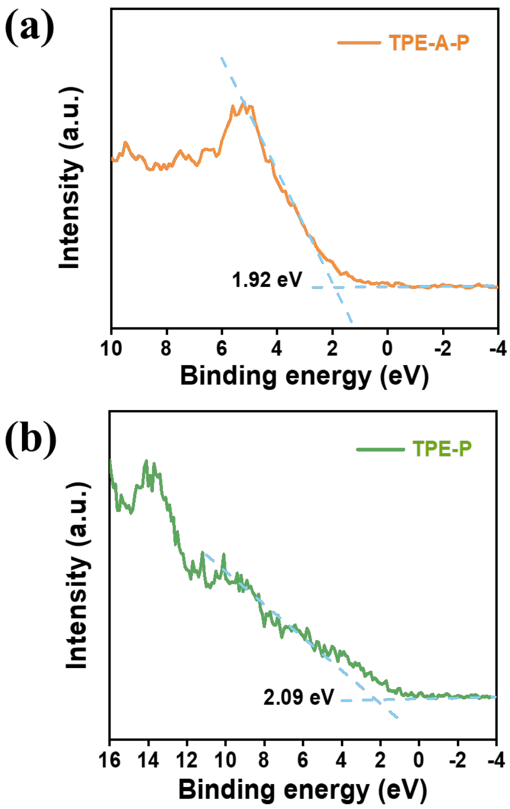

Figure S9. VB-XPS spectra of (a) TPE-A-P, (b) TPE-P.

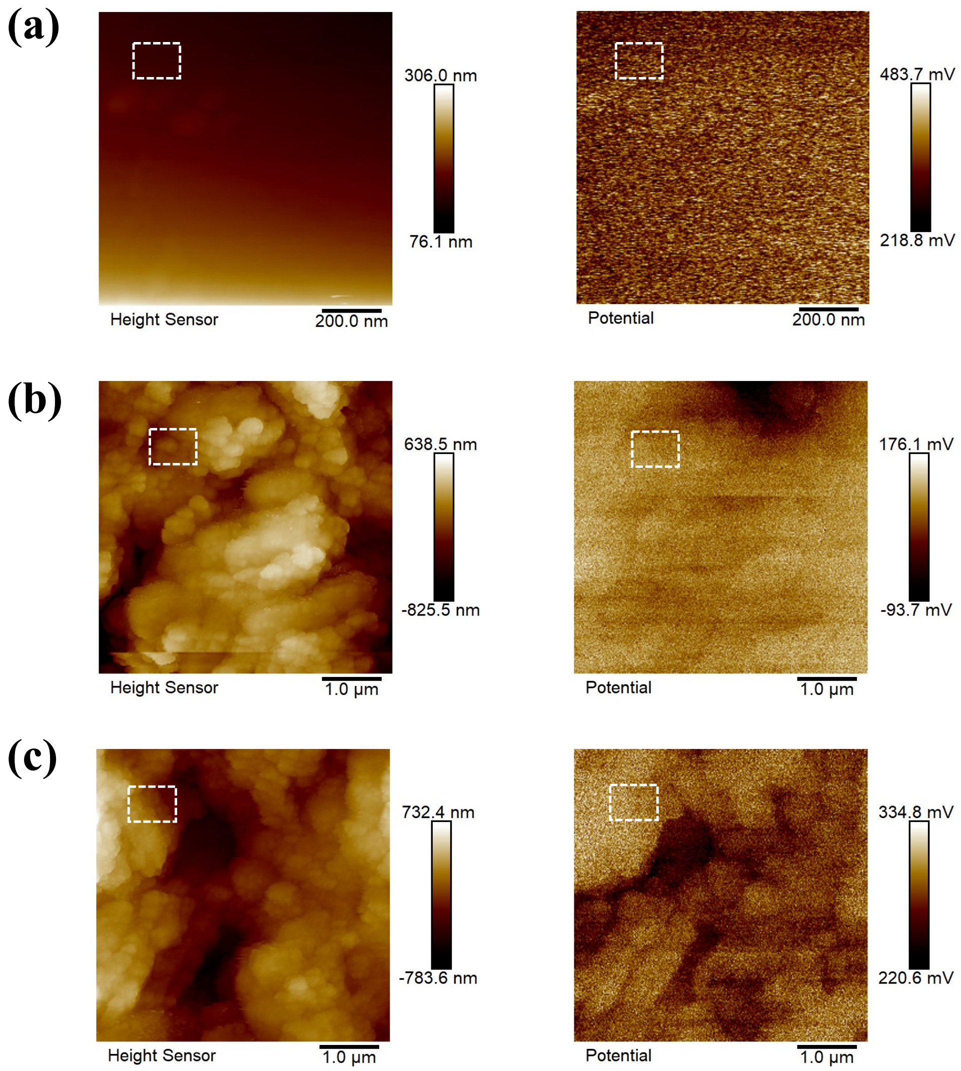

Figure S10. KPFM images of (a) HOPG, (b) TPE-A-P, (c) TPE-P.

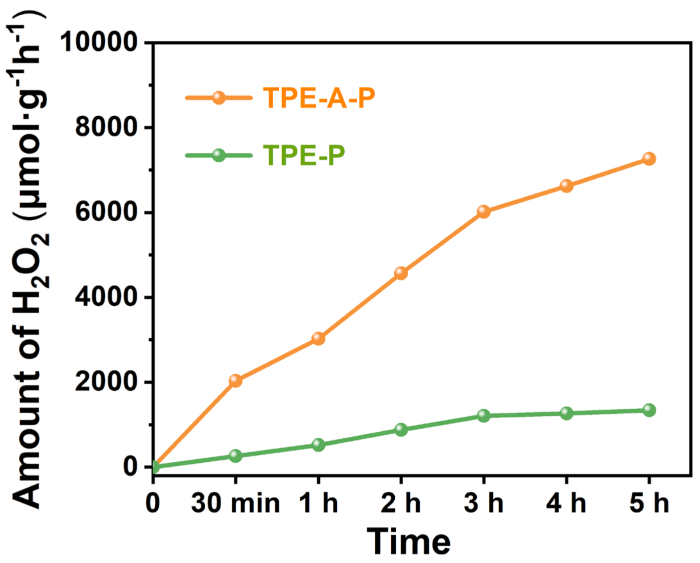

Figure S11. Photosynthetic amount of H_2O_2 generation from TPE-A-P and TPE-P in pure water under 5-hour irradiation.

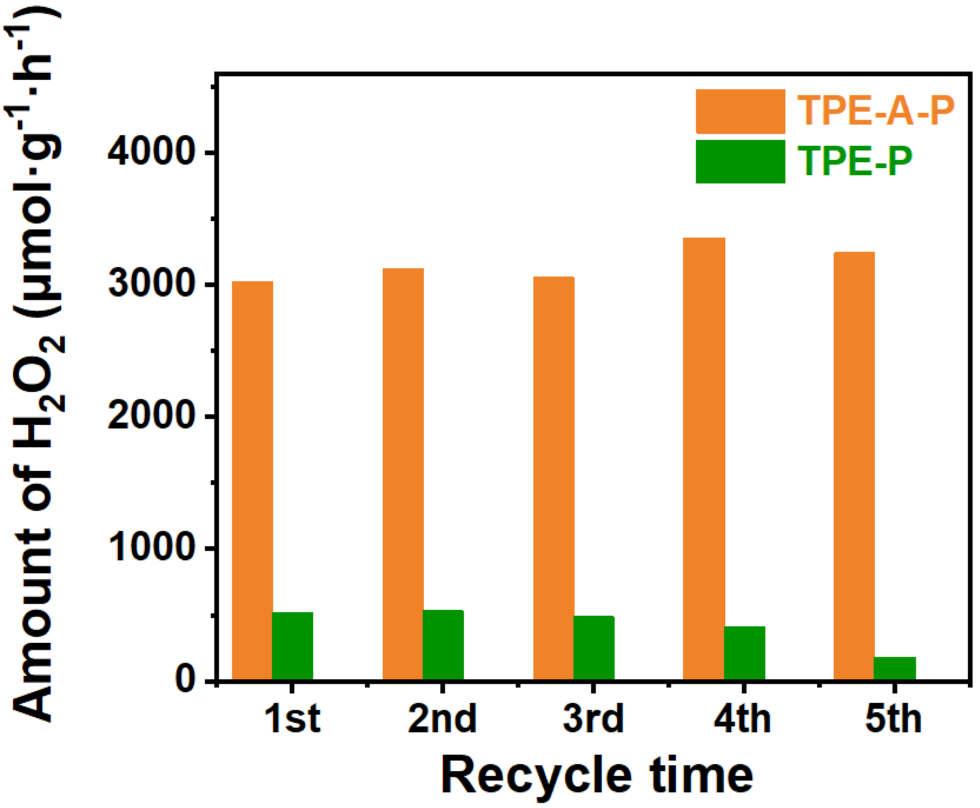

Figure S12. Cycle experiments of TPE-A-P and TPE-P in deionized water.

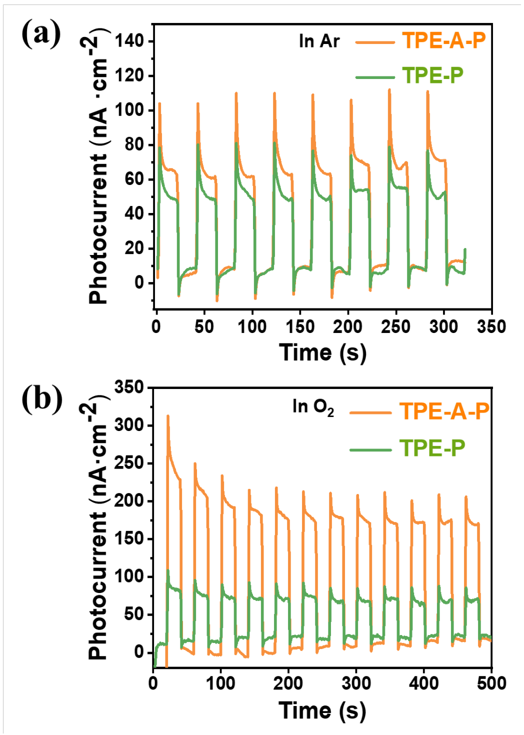

Figure S13. Current-Time (I-t) curves under visible light illumination of (a) TPE-A-P and TPE-P in Ar, (b) TPE-A-P and TPE-P in O_2.

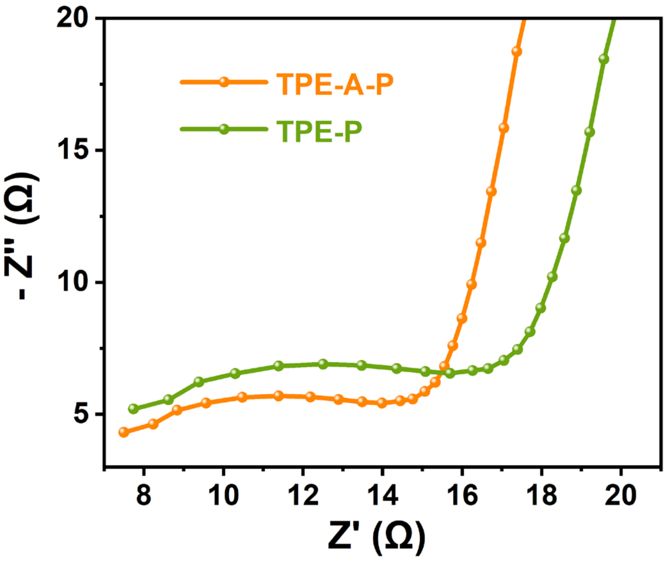

Figure S14. Nyquist plot of TPE-A-P and TPE-P.

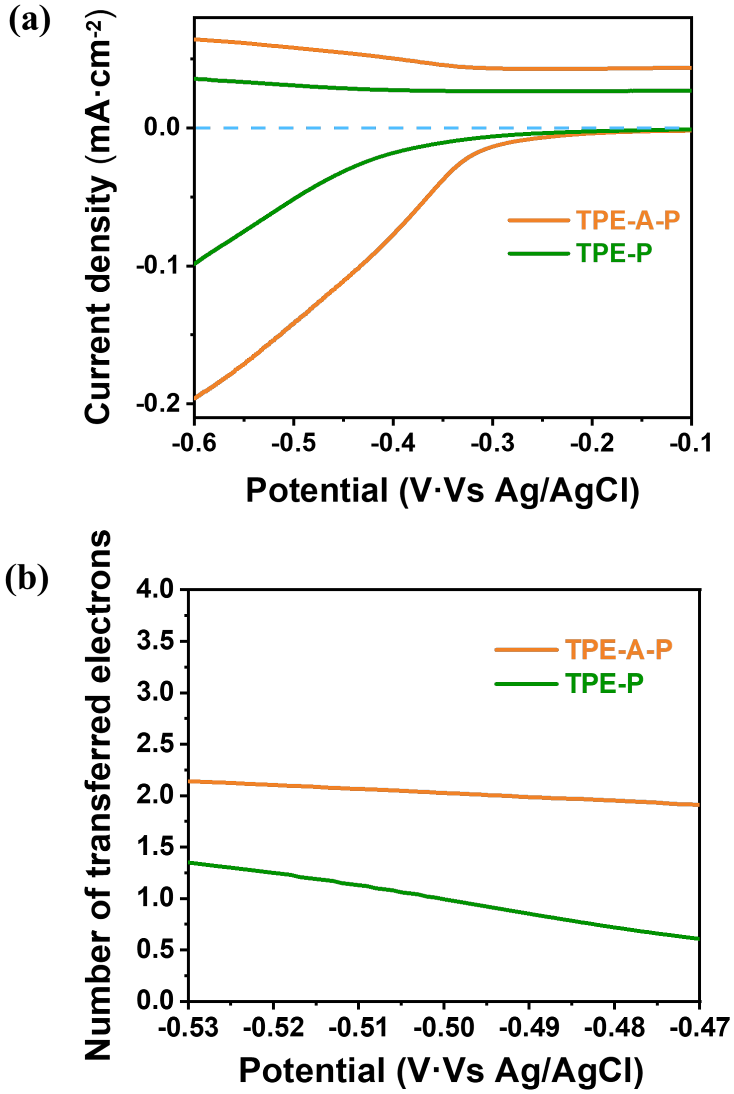

Figure S15. (a) ORR polarization curves of photocatalysts on RRDE at 1600 rpm in O_2-saturated phosphate buffer solution. (b) The corresponding number (n) of electron transfer as a function of the applied potential.

(a)

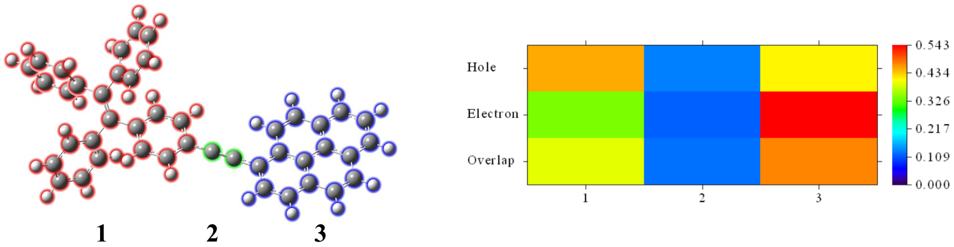

(b)

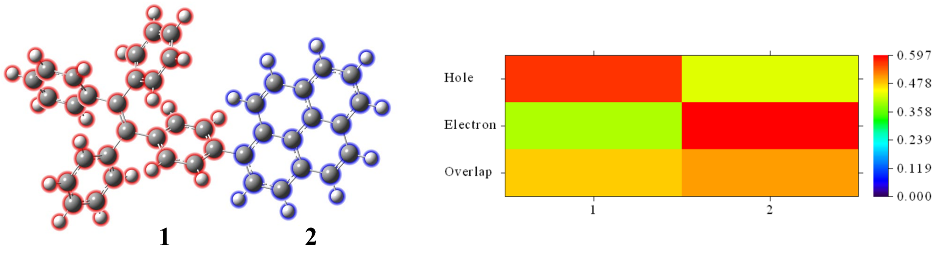

Figure S16. The distribution of electrons and holes in excited state of (a) TPE-A-P and (b) TPE-P.

Table S1. The efficiency comparison between TPE-A-P and previous reported photocatalysts for H_2O_2 production in anaerobic conditions.

Photocatalyst	Irradiation condition	solvent	H_2O_2 production rate ($\mu mol\ g^{-1}\ h^{-1}$)	reference
DE7-M	Solar Simulator	Water	None in N_2	[16]
BBTz	AM 1.5 G	Water	None in N_2	[17]
AQTEE-COP	$\lambda \geq 400$ nm	Water	None in N_2	[18]
TPT-Cz-phCN	$\lambda > 400$ nm	Water	1365 in Ar	[19]
HEP-TAPT-COF	$\lambda > 400$ nm	Water	None in Ar	[20]
SonoCOF-F2	Solar Simulator	Water	44 in N_2	[21]
TD-COF	400-700 nm	Water	1080 in Ar	[22]
NMT400	AM 1.5 G	Water: ethanol= 9:1	190 in N_2	[23]
TaptBtt	$\lambda > 420$ nm	Water	100 in Ar	[24]
DMCR-1NH	$\lambda > 420$ nm	Water	63 in N_2	[25]
TPE-A-P	$\lambda > 400$ nm	Water	1770 in Ar	**This wor**k

Table S2. Oscillator strengths of excited states of monomers from TPE-A-P and TPE-P.

Excited states	Oscillator strengths	
	TPE-A-P	TPE-P
$S_0 \rightarrow S_1$	1.4074	0.607
$S_0 \rightarrow S_2$	0.1294	0.1083
$S_0 \rightarrow S_3$	0.2442	0.4484
$S_0 \rightarrow S_4$	0.0485	0.0027
$S_0 \rightarrow S_5$	0.0618	0.0165

References

1 Frisch, M.J.; et al. Gaussian 09 (Gaussian, Inc, Wallingford, CT, 2009).

2 Grimme, S.; Antony, J.; Ehrlich, S.; Krieg, H.; A consistent and accurate ab initio parametrization of density functional dispersion correction (DFT-D) for the 94 elements H-Pu. *J. Chem. Phys*. **2010**, *132*, 154104.

3 Dennington, R.; et al. GaussView (Version 6.1.1, Shawnee, Mission, KS, 2019).

4 Adamo, C.; Barone, V. Toward reliable density functional methods without adjustable parameters: The PBE0 model. *J. Chem. Phys*. **1999**, *110*, 6158–6170.

5 Zheng, J.; Xu, X.; Truhlar, D.G. Minimally augmented Karlsruhe basis sets. *Theor. Chem. Acc.* **2011**, *128*, 295–305.

6 Papajak, E.; Zheng, J.; Xu, X.; H Leverentz,.R.; Truhlar, D.G. Perspectives on basis sets beautiful: seasonal plantings of diffuse basis functions. *J. Chem. Theory Comput.* **2011**, *7*, 3027–3034.

7 Yanai, T.; Tew, D.P.; Handy, N.C. A new hybrid exchange–correlation functional using the coulomb-attenuating method (CAM-B3LYP). *Chem. Phys. Lett*. **2004**, *393*, 51–57.

8 Francl, M.M.; et al. Self-consistent molecular orbital methods. XXIII. A polarization-type basis set for second-row elements. *J. Chem. Phys.* **1982**, *77*, 3654–3665.

9 Ditchfield, R.; Hehre, W.J.; Pople, J.A.; Self-consistent molecular-orbital methods. IX. An extended Gaussian-type basis for molecular-orbital studies of organic molecules. *J. Chem. Phys.* **1971**, *54*, 724–728.

10 Lu, T.; Chen, F. Multiwfn: a multifunctional wavefunction analyzer. *J. Comput. Chem.* **2012**, *33*, 580–592.

11 Lu, T.; Chen, F.-W. Comparison of computational methods for atomic charges. *Acta Phys.-Chim. Sin.* **2012**, *28*, 1–18.

12 Zhu, L.; et al. Small exciton binding energies enabling direct charge photogeneration towards low-driving-force organic solar cells. *Angew. Chem. Int. Ed.* **2021**, *60*, 15348–15353.

13 Lu, T.; Chen, F.-W. Quantitative analysis of molecular surface based on improved Marching Tetrahedra algorithm. *J. Mol. Graph.* **2012**, *38*, 314–323.

14 Zhang, J.; Lu, T. Efficient evaluation of electrostatic potential with computerized optimized code. *Phys. Chem. Chem. Phys.* **2021**, *23*, 20323–20328.

15 Liu, Z.; Lu, T.; Chen, Q. Intermolecular interaction characteristics of the all-carboatomic ring, cyclo[18]carbon: focusing on molecular adsorption and stacking. *Carbon* **2021**, *171*, 514–523.

16 Liu, L.J.; Gao, M.-Y.; Yang, H.F.; Wang, X.Y.; Li, X.B.; Cooper, A.I. Linear conjugated polymers for solar-driven hydrogen peroxide production: the importance of catalyst stability. *J. Am. Chem. Soc.* **2021**, *143*, 19287−19293.

17 Cheng, J.Z.; Wan, S.J.; Cao, S.W. Promoting solar-driven hydrogen peroxide production over thiazole-based conjugated polymers via generating and converting singlet oxygen. *Angew. Chem. Int. Ed.* **2023**, *62*, e202310476.

18 Xu, X.H.; Sa, R.J.; Huang, W.; Sui, Y.; Chen, W.T.; Zhou, G.Y.; Li, X.D.; Li, Y.T.; Zhong, H. Conjugated organic polymers with anthraquinone redox centers for efficient photocatalytic hydrogen peroxide production from water and oxygen under visible light irradiation without any additives. *ACS Catal.* **2022**, *12*, 12954−12963.

19 Yan, H.J.; et al. Enhancing photosynthesis efficiency of hydrogen peroxide by modulating side chains to facilitate water oxidation at low-energy barrier sites. *Adv. Mater.* **2024**, 2311535.

20 Chen, D.; Chen, W.; Wu, Y.; Wang, L.; Wu, X.; Xu, H.; Chen, L. Covalent organic frameworks containing dual O_2 reduction centers for overall photosynthetic hydrogen peroxide production. *Angew. Chem. Int. Ed.* **2023**, *62*, e202217479.

21 Zhao, W.; Yan, P.Y.; Li, B.Y.; Bahri, M.; Liu, L.J.; Zhou, X.; Clowes, R.; Browning, N. D.; Wu, Y.; Ward, J. W.; Cooper, A.I. Accelerated synthesis and discovery of covalent organic framework photocatalysts for hydrogen peroxide production. *J. Am. Chem. Soc.* **2022**, *144*, 9902–9909.

22 Yue, J.; Song, L.; Fan, Y.F.; Pan, Z.X.; Yang, P.; Ma, Y.; Xu, Q.; Tang, B. Thiophene-containing covalent organic frameworks for overall photocatalytic H_2O_2 synthesis in water and seawater. *Angew. Chem. Int. Ed.* **2023**, *62*, e202309624.

23 Yang, C.; Wan, S.J.; Zhu, B.C.; Yu, J.G.; Cao, S.W. Calcination-regulated microstructures of donor-acceptor polymers towards enhanced and stable photocatalytic H_2O_2 production in pure water. *Angew. Chem. Int. Ed*. **2022**, *61*, e202208438.

24 Qin, C.C.; Wu, X.D.; Tang, L.; Chen, X.H.; Li, M.; Mou, Y.; Su, B.; Wang, S.B.; Feng, C.Y.; Liu, J.W.; Yuan, X.Z.; Zhao, Y.L.; Wang, H. Dual donor-acceptor covalent organic frameworks for hydrogen peroxide photosynthesis. *Nat Commun*. **2023**, *14*, 5238.

25 Das, P.; Chakraborty, G.; Roeser, J.; Vogl, S.; Rabeah, J.; Thomas, A. Integrating bifunctionality and chemical stability in covalent organic frameworks via one-pot multicomponent reactions for solar-driven H_2O_2 production. *J. Am. Chem. Soc*. **2023**, *145*, 2975–2984.
